# Use of the ankle-brachial index combined with the percentage of mean arterial pressure at the ankle to improve prediction of all-cause mortality in type 2 diabetes mellitus: an observational study

**DOI:** 10.1186/s12933-020-01149-7

**Published:** 2020-10-09

**Authors:** Yu-Hsuan Li, Wayne Huey-Herng Sheu, I-Te Lee

**Affiliations:** 1 Division of Endocrinology and Metabolism, Department of Internal Medicine, Taichung Veterans General Hospital, No. 1650 Taiwan Boulevard, Section 4, Taichung, 40705 Taiwan; 2 School of Medicine, National Yang-Ming University, Taipei, 11221 Taiwan; 3 Rong Hsing Research Center For Translational Medicine, College of Life Sciences, National Chung Hsing University, Taichung, Taiwan; 4 School of Medicine, Chung Shan Medical University, Taichung, 40201 Taiwan; 5 College of Science, Tunghai University, Taichung, 40704 Taiwan

**Keywords:** Ankle-brachial index, Diabetes, Lower extremity artery disease, Mortality, Percentage of the mean arterial pressure, Peripheral artery disease

## Abstract

**Background:**

Peripheral artery disease (PAD) in the lower extremities is a common complication of type 2 diabetes and has been shown to be associated with mortality. The ankle-brachial index (ABI) is a simple noninvasive method to screen PAD, but this method has limited sensitivity. We hypothesized that using the percentage of mean arterial pressure (%MAP) in combination with the ABI would improve the prediction of mortality.

**Methods:**

We retrospectively collected data from patients with type 2 diabetes who had undergone ABI and  %MAP measurements at our hospital. We separated the cohort into four groups according to their ABI and  %MAP values, and we examined whether these indices were associated with mortality.

**Results:**

A total of 5569 patients (mean age, 65 ± 11 years) were enrolled. During the follow-up period (median, 22.9 months), 266 (4.8%) of the enrolled patients died. The combination of ABI and  %MAP was significantly more effective than ABI alone for predicting mortality (C index of 0.62, 95% confidence interval [CI] of 0.57 to 0.65 vs. C index of 0.57, 95% CI of 0.53 to 0.62; P = 0.038). In multivariate analysis (with a reference group defined by ABI > 0.90 and  %MAP ≤ 45%), the highest risk of mortality was seen in patients with ABI ≤ 0.90 and  %MAP > 45% (hazard ratio = 2.045 [95% CI 1.420, 2.945], P < 0.001).

**Conclusions:**

The use of  %MAP alongside ABI appears to significantly improve the prediction of all-cause mortality in patients with type 2 diabetes.

## Background

Diabetes mellitus (DM) has become a heavy health burden because of its high global prevalence [1]. Peripheral artery disease (PAD) of the lower extremities is a common complication of type 2 DM [2]. The mortality risk of PAD was reported to be between those of myocardial infarction and stroke in a cohort study with a mean follow-up of 5.9 years, and DM increased the risk of mortality by 1.4 times in patients with PAD [3]. Although PAD is clinically confirmed using noninvasive angiography (i.e., computed tomography angiography and magnetic resonance angiography) or invasive angiography for anatomical assessments, the use of the ankle-brachial index (ABI) as a simple screen for PAD in patients with DM and cardiovascular risk is recommended by the American Heart Association/American College of Cardiology (AHA/ACC) guidelines on the management of patients with lower extremity PAD [4].

However, the commonly used ABI value of < 0.90 has been reported to have only 75% sensitivity for PAD diagnosis, and the sensitivity is even lower in patients with DM than in those without DM [5, 6]. Since borderline low ABI values between 0.91 and 0.99 are associated with a higher risk of PAD and mortality than ABI ≥ 1.00 [7, 8], it has been suggested that sensitivity could be increased by raising the cutoff value for normal ABI to 1.00 [5, 9]. However, the specificity is markedly decreased when the cutoff value for normal ABI is raised [10]. Instead, an increase in diagnostic accuracy could be achieved by using other parameters in combination with ABI [5].

The combination of ABI and percentage of mean arterial pressure (%MAP) at the ankle has been reported to show better diagnostic accuracy than ABI alone [10]. The  %MAP can be calculated from a pulse volume recording at the ankle and automatically reported by the ABI-measuring machine, and so it is a convenient index to use along with the ABI in screening for PAD [10–12]. A previous study has shown that  %MAP > 45% predicts a high mortality risk in patients with normal ABI [13].

In summary,  %MAP is a useful biomarker and can be conveniently measured to detect PAD in patients with normal ABI, especially in patients with DM, for whom the sensitivity of ABI for PAD diagnosis is relatively low. However, the utility of  %MAP in combination with ABI in predicting long-term mortality is still not clear in patients with type 2 DM. Therefore, in this study, we aimed to determine whether the combination of low ABI and high  %MAP would be a better predictor of all-cause mortality than low ABI alone in patients with type 2 DM.

## Materials and methods

### Study design and population

This retrospective cohort study was conducted at Taichung Veterans General Hospital in Taiwan. According to our computer-interpretable guidelines from August 2016, ABI was suggested via the annual diabetes review program of the hospital information system if ABI data had not been available in patients who were older than 50 years and had participated in the diabetes pay-for performance (P4P) program [14].

From the hospital database, we retrospectively identified all patients with DM who had undergone ABI assessment between August 01, 2016 and July 31, 2019. Moreover, each enrolled patient was required to fulfill at least one of the following inclusion criteria: (1) age ≥ 50 years, (2) diabetes duration ≥ 10 years, (3) current smoking, (4) a history of cardiovascular disease (CVD), (5) hypertension, (6) body mass index (BMI) ≥ 27 kg/m^2^, (7) hemoglobin A1c (HbA1c) ≥ 7%, (8) total cholesterol ≥ 160 mg/dL (4.1 mmol/L), (9) high-density lipoprotein (HDL) cholesterol < 50 mg/dL (1.29 mmol/L) in women or < 40 mg/dL (1.03 mmol/L) in men, (10) triglycerides ≥ 150 mg/dL (1.69 mmol/L), (11) estimated glomerular filtration rate (eGFR) < 60 mL/min/1.73 m^2^, or (12) albuminuria. We excluded patients who met any of the following conditions: (1) incomplete laboratory data; (2) not type 2 DM; (3) incomplete ABI, brachial-ankle pulse wave velocity (baPWV), or  %MAP data due to lack of a complete four-limb assessment; (4) unreliable ABI data due to previous lower-limb surgery, pregnancy, or hemodialysis treatment; or (5) ABI > 1.40.

ABI measurements were made using a validated device (VP-1000 Plus; Omron Healthcare Co. Ltd., Kyoto, Japan). After patients had rested in a supine position for at least 5 min, cuffs which were connected to both a plethysmographic sensor for detecting volume change and an oscillometric pressure sensor for detecting blood pressure were placed on the brachia and ankles. In addition to ABI, this device automatically reports the baPWV and  %MAP of the ankle pulse volume waveform. These data, along with anthropometric data and results of laboratory tests performed within 3 months of the ABI assessment, were extracted from the electronic medical records. For patients who had undergone repeated assessments during this period, only the data from the first assessment were recorded. This research protocol was approved by the Institutional Review Board of Taichung Veterans General Hospital, and the need for informed consent was waived.

### Assessments

The following laboratory data were recorded: total cholesterol, HDL cholesterol, triglycerides, glucose, HbA1c, and creatinine. The eGFR was calculated using the Modification of Diet in Renal Disease equation, i.e., eGFR = 186 × (serum creatinine [mg/dL])^−1.154^ × (age [years])^−0.203^ (× 0.742, if female) [15]. The urinary albumin-to-creatinine ratio (UACR) was calculated using the formula UACR = albumin (mg)/creatinine (g), and albuminuria was defined as UACR ≥ 300 mg/g [15]. Hypertension was defined as systolic blood pressure ≥ 140 mmHg, diastolic blood pressure ≥ 90 mmHg, or current use of an antihypertensive drug.

The  %MAP value was automatically determined based on the ankle pulse volume waveform during ABI measurement. The ABI was calculated as (systolic pressure of the ankle in each leg)/(the higher of the two arm systolic pressures) [16]. The  %MAP was calculated as (average amplitude of pulse wave)/(maximal amplitude of pulse wave) × 100%, and the average amplitude was calculated by dividing the area between a pulse volume curve and its initial base by duration of a pulse wave [12, 17]. The baPWV was calculated as (distance from the suprasternal notch to the brachium ‒ distance from the suprasternal notch to the ankle)/(the time interval between waves detected at the brachium and ankle), where the distances from the suprasternal notch to the brachium and ankle were estimated based on the patient’s body height [18]. The reproducibility of the ABI and  %MAP has been demonstrated in our previous study [19], and the 95% confidence interval (CI) between repeated measurements of baPWV was 4.95 ± 46.46 cm/sec based on Bland–Altman plots. The lower ABI value and the higher  %MAP and baPWV values between lower limbs in an individual were used for the analyses. ABI ≤ 0.90 and  %MAP > 45% were defined as abnormal [13, 19].

### Statistical analysis

Continuous data were summarized as the mean ± standard deviation; differences among four study subgroups were analyzed using one-way analysis of variance, and the Scheffe post hoc test was conducted to examine the differences between the high  %MAP and normal  %MAP subgroups in patients with a normal ABI group or a low ABI group. Categorical data were summarized as numbers with percentages (%) and compared among groups using the Chi square test. Correlation was assessed using the Pearson correlation coefficient (*r*). The primary endpoint was all-cause mortality. Information on deaths registered up to August 31, 2019 was obtained from the Ministry of Health and Welfare, Executive Yuan, Taiwan. Causes of death were categorized according to the diagnosis codes of the International Classification of Disease 10, Clinical Modification.

The improvement in mortality prediction caused by considering the  %MAP along with the ABI was assessed by examining the increases in the area under the receiver operating characteristic curve (AUC). The performance of the model containing both ABI and  %MAP compared to the model with ABI alone was evaluated by the C index. The integrated discrimination improvement (IDI) and continuous net reclassification improvement (NRI) were also assessed to quantify the improvement in predictive ability by adding  %MAP.

The cumulative risk of the all-cause mortality was assessed using Kaplan–Meier analysis; the log-rank test was used to determine whether the differences between groups were significant. Multivariable Cox proportional hazards regression analysis was conducted to identify the independent predictors of mortality; hazard ratio (HR) and 95% CI were calculated. A two-sided P value < 0.05 was considered statistically significant. Statistical analysis was performed using SPSS v22.0 (IBM Corp., Armonk, NY, USA) and R software v3.4.

## Results

**Fig. 1:**
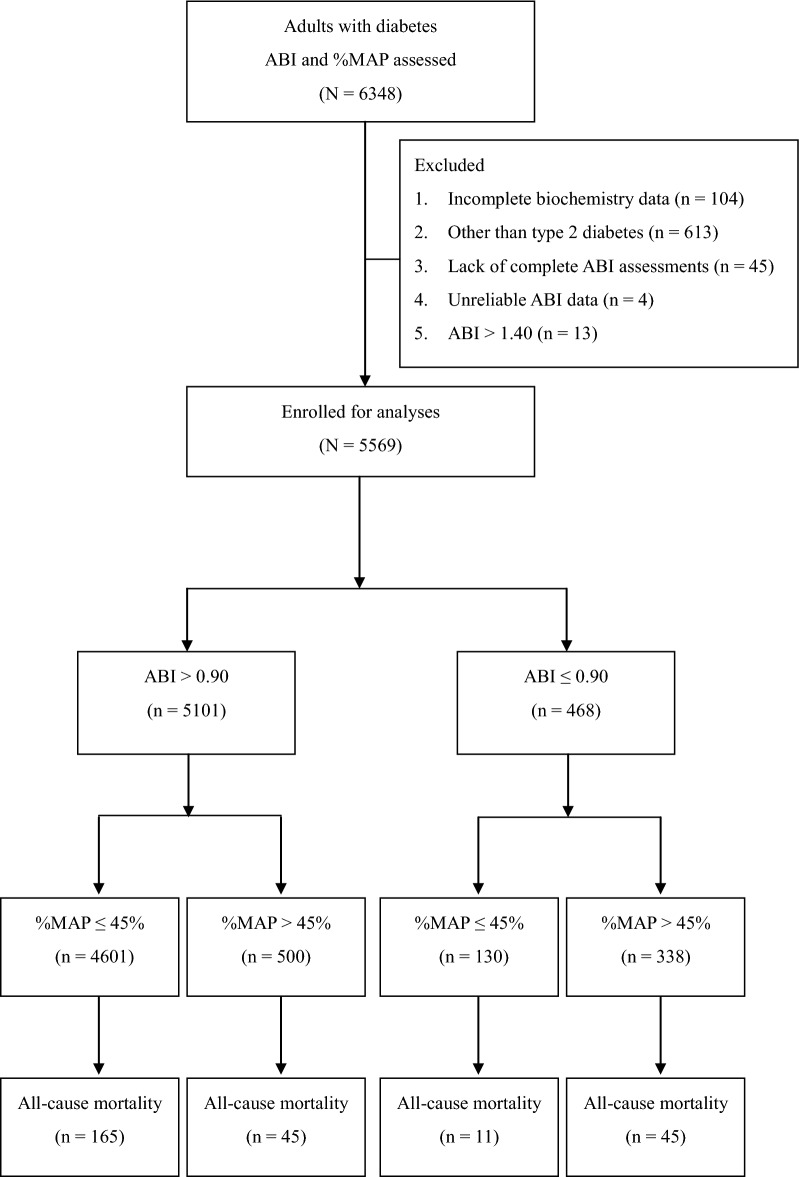
Study flow diagram. ( *%MAP* percentage of mean arterial pressure, *ABI* ankle-brachial index)

A total of 5569 patients were enrolled in this study, and  %MAP was inversely correlated with ABI (*r* = − 4.70, P < 0.001). Based on the ABI value, all patients were first separated into two groups: a normal ABI group and a low ABI group. Each group was then separated into two subgroups according to the  %MAP value. Thus, there were four subgroups: patients with normal ABI and normal  %MAP (n = 4601); patients with normal ABI but high  %MAP (n = 500); patients with low ABI but normal  %MAP (n = 130); and patients with low ABI and high  %MAP (n = 338, Fig. 1).

Table 1 shows the baseline characteristics of patients in the different subgroups. Patients with high  %MAP were significantly older than patients with normal  %MAP in both the normal ABI group (70 ± 12 vs. 64 ± 10 years, P < 0.001) and the low ABI group (73 ± 12 vs. 65 ± 12 years, P < 0.001). The baPWV was significantly higher in the high  %MAP subgroup than in the normal  %MAP subgroup in both the normal ABI group (P < 0.001) and the low ABI group (P < 0.001). BMI, ABI and eGFR were significantly lower in the high  %MAP subgroup than in the normal  %MAP subgroup in both the normal ABI group (P = 0.027, P < 0.001, and P < 0.001; respectively) and the low ABI group (all P values < 0.001). The prevalence rates of CVD, albuminuria and use of antiplatelet drugs were significantly higher in the high  %MAP subgroup than in the normal  %MAP subgroup in both the normal ABI group (all P values < 0.001) and the low ABI group (P = 0.015, P = 0.004, and P < 0.001; respectively). The proportion of patients using oral antihyperglycemic drugs was significantly lower in the high  %MAP subgroup than in the normal  %MAP subgroup in both the normal ABI group (P < 0.001) and the low ABI group (P = 0.008). Patients with high  %MAP had a significantly higher proportion of females, a higher prevalence of hypertension, higher systolic blood pressure, higher prevalence rates of antihypertensive drug use and insulin therapy, and longer diabetes duration than those with normal  %MAP in the normal ABI group (all P < 0.001), but the same was not true in the low ABI group.

**Table 1:** Characteristics of enrolled patients categorized based on a combination of ABI and  %MAP*

Group	Normal ABI (n = 5101)			Low ABI (n = 468)			P^#^
Subgroup	Normal % MAP	High %MAP	P^†^	Normal %MAP	High %MAP	P^‡^	
	(n = 4601)	(n = 500)		(n = 130)	(n = 338)		
	Mean ± SD	Mean ± SD		Mean ± SD	Mean		
Age (year)	64 ± 10	70 ± 12	< 0.001	65 ± 12	73 ± 12	< 0.001	< 0.001
Male, n (%)	2559 (55.6%)	203 (40.6%)	< 0.001	66 (50.8%)	196 (58.0%)	0.192	< 0.001
Diabetes duration (year)	11 ± 7	14 ± 8	< 0.001	14 ± 8	15 ± 8	0.4	< 0.001
Currently smoking, n (%)	415 (9.0%)	32 (6.4%)	0.06	11 (8.5%)	26 (7.7%)	0.932	0.197
CVD history, n (%)	357 (7.8%)	72 (14.4%)	< 0.001	22 (16.9%)	96 (28.4%)	0.015	< 0.001
BMI (kg/m^2^)	25.9 ± 4.1	25.3 ± 4.2	0.027	28.0 ± 5.0	25.0 ± 4.0	<0.001	< 0.001
Systolic BP (mmHg)	135 ± 19	144 ± 24	< 0.001	140 ± 20	145 ± 25	0.139	< 0.001
Diastolic BP (mmHg)	77 ± 12	76 ± 13	0.519	75 ± 15	74 ± 16	0.584	< 0.001
Fasting glucose (mmol/L)	8.2 ± 3.6	8.2 ± 3.4	0.999	8.3 ± 2.9	8.7 ± 3.8	0.76	0.066
HbA1c (%)	7.5 ± 1.6	7.6 ± 1.6	0.99	7.9 ± 2.0	7.8 ± 1.9	0.997	0.001
Total cholesterol (mmol/L)	4.1 ± 0.9	4.1 ± 1.0	0.966	4.1 ± 0.9	4.0 ± 1.0	0.881	0.188
HDL cholesterol (mmol/L)	1.3 ± 0.4	1.3 ± 0.4	0.999	1.2 ± 0.3	1.2 ± 0.3	0.915	< 0.001
Triglycerides (mmol/L)	1.6 ± 1.3	1.5 ± 1.2	0.637	2.0 ± 1.9	1.7 ± 1.1	0.193	0.004
eGFR (mL/min/1.73 m^2^)	81 ± 28	70 ± 34	< 0.001	73 ± 34	53 ± 32	< 0.001	< 0.001
UACR ≥ 300 mg/g	498 (10.8%)	88 (17.6%)	< 0.001	19 (14.6%)	94 (27.8%)	0.004	< 0.001
ABI	1.11 ± 0.09	1.07 ± 0.09	< 0.001	0.83 ± 0.08	0.68 ± 0.21	< 0.001	< 0.001
baPWV (cm/sec)	1823 ± 433	2087 ± 671	< 0.001	1867 ± 690	2176 ± 1143	< 0.001	< 0.001
Ankle %MAP (%)	39.4 ± 3.1	47.2 ± 1.8	< 0.001	40.9 ± 3.1	50.4 ± 3.5	< 0.001	< 0.001
Antiplatelet, n (%)	1212 (26.3%)	178 (35.6%)	< 0.001	64 (49.2%)	273 (80.8%)	< 0.001	< 0.001
Statins, n (%)	3253 (70.7%)	351 (70.2%)	0.855	97 (74.6%)	250 (74.0%)	0.979	0.446
Hypertension, n (%)	3466 (75.3%)	414 (82.8%)	< 0.001	115 (88.5%)	317 (93.8%)	0.081	< 0.001
Antihypertensive drugs, n (%)	2397 (52.1%)	307 (61.4%)	< 0.001	87 (66.9%)	257 (76.0%)	0.06	< 0.001
ACE inhibitors or ARBs, n (%)	1714 (37.3%)	213 (42.6%)	0.022	58 (44.6%)	168 (49.7%)	0.377	< 0.001
α-blockers, n (%)	255 (5.5%)	74 (14.8%)	< 0.001	11 (8.5%)	64 (18.9%)	0.009	< 0.001
β-blockers, n (%)	949 (20.6%)	138 (27.6%)	< 0.001	38 (29.2%)	135 (39.9%)	0.041	< 0.001
Calcium channel blockers, n (%)	217 (4.7%)	28 (5.6%)	0.443	12 (9.2%)	37 (10.9%)	0.708	< 0.001
Diuretics, n (%)	396 (8.6%)	88 (17.6%)	< 0.001	24 (18.5%)	95 (28.1%)	0.043	< 0.001
Insulin therapy, n (%)	1032 (22.4%)	159 (31.8%)	< 0.001	42 (32.3%)	132 (39.1%)	0.213	< 0.001
Oral antihyperglycemic drugs	4175 (90.7%)	424 (84.8%)	< 0.001	113 (86.9%)	254 (75.1%)	0.008	< 0.001
Insulin secretagogues, n (%)	1638 (35.6%)	190 (38.0%)	0.311	45 (34.6%)	96 (28.4%)	0.23	0.028
Metformin, n (%)	1838 (39.9%)	174 (34.8%)	0.029	54 (41.5%)	69 (20.4%)	< 0.001	< 0.001
Thiazolidinediones, n (%)	2737 (59.5%)	287 (57.4%)	0.393	72 (55.4%)	191 (56.5%)	0.908	0.469
α-Glucosidase inhibitors, n (%)	530 (11.5%)	40 (8.0%)	0.022	17 (13.1%)	17 (5.0%)	0.005	< 0.001
DPP4 inhibitors, n (%)	1046 (22.7%)	95 (19.0%)	0.065	30 (23.1%)	51 (15.1%)	0.056	0.002
SGLT2 inhibitors, n (%)	415 (9.0%)	57 (11.4%)	0.096	8 (6.2%)	31 (9.2%)	0.384	0.212
Mortality, n (%)	165 (3.6%)	45 (9.0%)	< 0.001	11 (8.5%)	45 (13.3%)	0.197	< 0.001
CVD, n (%)	40 (0.9%)	19 (3.8%)		4 (3.1%)	27 (8.0%)		
Cancer, n (%)	87 (1.9%)	12 (2.4%)		2 (1.5%)	2 (0.6%)		
Others, n (%)	38 (0.8%)	14 (2.8%)		5 (3.8%)	16 (4.7%)		
Incidence of mortality (deaths/100 person-years)	2.0	5.0		4.8	8.3		

Continuous data are presented as the mean ± SD, and categorical data are presented as numbers (percentages)

*: low ABI was defined as an ABI value ≤ 0.90 and normal ABI > 0.90; high  %MAP was defined as a  %MAP > 45% and normal  %MAP ≤ 45%

^#^P: denotes a significant difference across the four subgroups

^†^P: post hoc comparison between two subgroups in patients with normal ABI; ^‡^P: post hoc comparison between two subgroups in patients with low ABI

*%MAP* percentage of mean arterial pressure, *ABI* ankle-brachial index, *ACE* angiotensin-converting enzyme, *ARB* angiotensin II receptor antagonist, *baPWV* brachial-ankle pulse wave velocity, *BMI* body mass index, *BP* blood pressure, *CVD* cardiovascular disease, *DPP4* dipeptidyl peptidase-4, *eGFR* estimated glomerular filtration rate, *HbA1c* hemoglobin A1c, *HDL* high-density lipoprotein, *SD* standard deviation, *SGLT2* sodium glucose cotransporter 2, *UACR* urine albumin-to-creatinine ratio

**Fig. 2:**
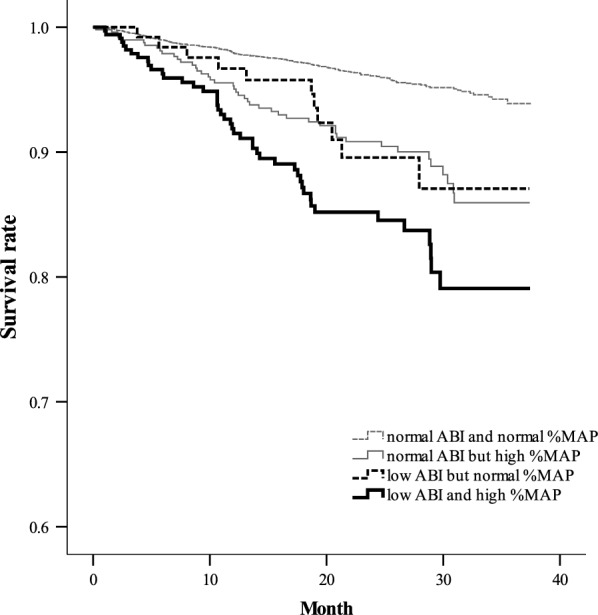
Kaplan-Meier curves showing the survival rates across the four groups, defined based on an ankle-brachial index (ABI) threshold of 0.90 and an ankle percentage of mean arterial pressure (%MAP) threshold of 45%

Over a median follow-up of 22.9 months (interquartile range: 13.2-29.7 months), 266 (4.8%) of the 5569 enrolled patients died. The incidence rates of mortality were 2.0 deaths/100 person-years in the normal ABI and normal  %MAP subgroup, 5.0 deaths/100 person-years in the normal ABI but high  %MAP subgroup, 4.8 deaths/100 person-years in the low ABI but normal  %MAP subgroup, and 8.3 deaths/100 person-years in the low ABI and high  %MAP subgroup, respectively; the survival rates were significantly different across these four subgroups (log-rank test P < 0.001, Fig. 2).

**Fig. 3:**
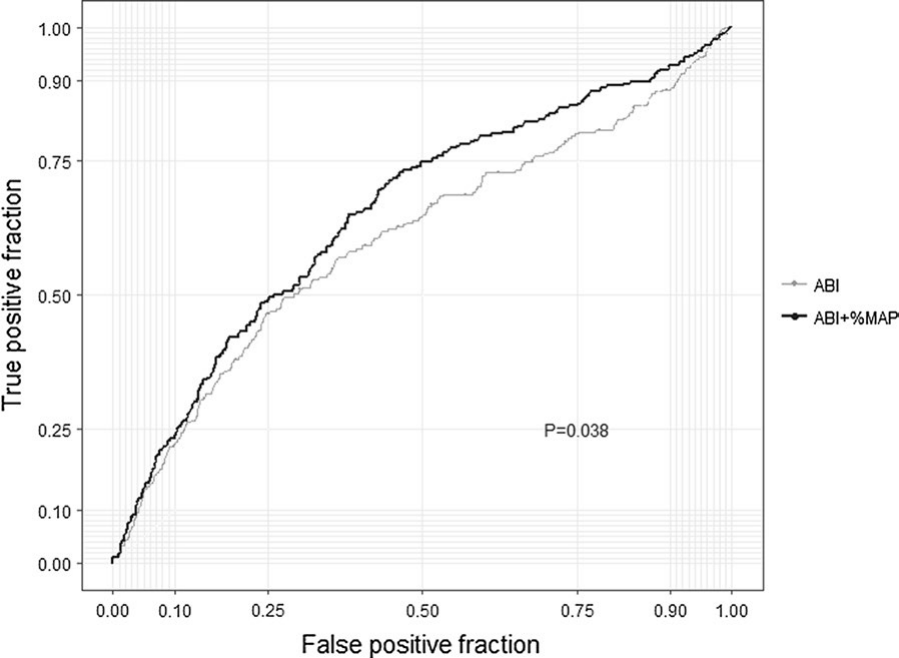
Receiver operating characteristic curves for prediction of all-cause mortality in the ABI alone model and in the ABI +  %MAP model (*%MAP* percentage of mean arterial pressure, *ABI* ankle-brachial index)

**Fig. 4:**
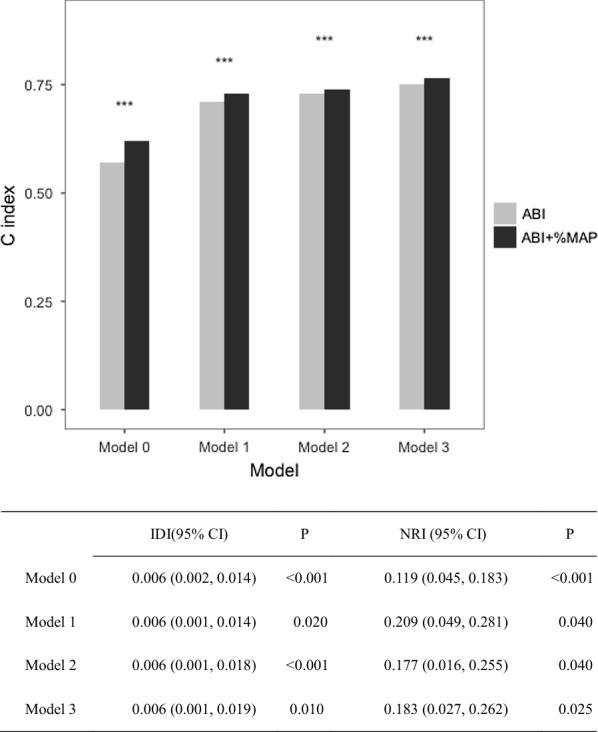
The C index, integrated discrimination improvement (IDI), and continuous net reclassification improvement (NRI) based on each different model: Model 0: no adjustment; Model 1: adjusted for age and sex; Model 2: adjusted for age, sex, diabetes duration, smoking, cardiovascular disease, and body mass index; Model 3: adjusted for age, sex, diabetes duration, smoking, cardiovascular disease, body mass index, hemoglobin A1c, total cholesterol, high-density lipoprotein cholesterol, triglycerides, estimated glomerular filtration rate, albuminuria, brachial-ankle pulse wave velocity, systolic blood pressure, use of antihypertensive drugs, use of insulin, use of statins, and use of antiplatelet agents

To evaluate how the inclusion of the  %MAP along with ABI affected the prediction of all-cause mortality, we analyzed the increases in the AUC. We used ABI as the standard risk factor; the AUC increased significantly from 0.57 (95% CI 0.53–0.62) for the ABI alone model to 0.62 (95% CI 0.57–0.65) for the ABI plus  %MAP model (P = 0.038; Fig. 3). Furthermore, the use of  %MAP along with ABI yielded a significant IDI of 0.006 (95% CI 0.002–0.014, P < 0.001) and a significant NRI of 0.119 (95% CI 0.045-0.183, P < 0.001). The predictive model with  %MAP and ABI was still significantly better than the model with ABI alone after adjusting for age, sex, diabetes duration, smoking, CVD history, BMI, hemoglobin A1c, total cholesterol, HDL cholesterol, triglycerides, eGFR, albuminuria, baPWV, systolic blood pressure, use of antihypertensive drugs, use of insulin, use of statins, and use of antiplatelet agents (Fig. 4).

Multivariate Cox regression analysis was performed using the patients with normal ABI and normal  %MAP as the reference group. The highest risk for mortality was observed in patients with low ABI and high  %MAP (HR = 2.045, 95% CI 1.420 to 2.945, P < 0.001), followed by patients with low ABI but normal  %MAP (HR = 1.730, 95% CI 0.932 to 3.209) and patients with normal ABI but high  %MAP (HR = 1.579, 95% CI 1.120 to 2.226). Furthermore, high  %MAP was found to be a significant predictor of all-cause mortality in patients with normal ABI (P = 0.009, Table 2).

**Table 2:** Cox regression analysis for all-cause mortality*

	Crude				Model 1				Model 2				Model 3			
	HR	95% CI		P	HR	95% CI		P	HR	95% CI		P	HR	95% CI		P
Normal ABI and normal %MAP^#^	1.000				1.000				1.000				1.000			
Normal ABI but high %MAP	2.510	(1.805, 3.490)		< 0.001	2.231	(1.597, 3.116)		< 0.001	2.018	(1.443, 2.823)		< 0.001	1.579	(1.120, 2.226)		0.009
Low ABI but normal %MAP	2.390	(1.298, 4.400)		0.005	2.171	(1.178, 4.000)		0.013	1.965	(1.063, 3.631)		0.031	1.730	(0.932, 3.209)		0.082
Low ABI and high %MAP	4.167	(2.997, 5.795)		< 0.001	3.076	(2.199, 4.305)		< 0.001	2.539	(1.801, 3.578)		< 0.001	2.045	(1.420, 2.945)		< 0.001

* The cumulative mortality rate was 4.8% in a total of 5569 enrolled patients

^#^Low ABI was defined as an ABI value ≤ 0.90 and normal ABI > 0.90; high  %MAP was defined as  %MAP > 45% and normal  %MAP ≤ 45%

*%MAP* percentage of mean arterial pressure, *ABI* ankle-brachial index, *CI* confidence interval, *HR* hazard ratio

Model 1: adjusted for age and sex; Model 2: adjusted for age, sex, diabetes duration, smoking, cardiovascular disease, and body mass index; Model 3: adjusted for age, sex, diabetes duration, smoking, cardiovascular disease, body mass index, hemoglobin A1c, total cholesterol, high-density lipoprotein cholesterol, triglycerides, estimated glomerular filtration rate, albuminuria, brachial-ankle pulse wave velocity, systolic blood pressure, use of antihypertensive drugs, use of insulin, use of statins, and use of antiplatelet agents

## Discussion

The main finding of our study was that high ankle  %MAP acted synergistically with low ABI to improve the prediction of all-cause mortality in patients with type 2 DM. When these two indices were used in combination, ABI ≤ 0.90 and  %MAP > 45%, predicted an approximately two-fold higher mortality risk than ABI > 0.90 and  %MAP ≤ 45%. These results corroborate our previous study, which showed that high  %MAP was a significant predictor of all-cause mortality [13]. The strength of the present study is that we demonstrated the synergy of ABI and  %MAP for the prediction of mortality in a large sample of more than 5000 patients with type 2 DM. Furthermore, we used NRI and IDI to quantify the improvement of prediction. Because of the limitation to use only AUC or C-index for the risk prediction model, NRI and IDI were recommended to give complementary information for model performance [20]. Thus, a combination of  %MAP and ABI appears to be more effective than ABI alone in predicting the risk of death, and this finding is important because global mortality from PAD is continually increasing [21].

PAD is associated with several atherosclerotic morbidities and is predictive of long-term cardiovascular events [3]. An occluded artery in a lower extremity will result in decreased blood flow, reflected by a decrease in systolic blood pressure and a flattened pulse volume waveform at the ankle [16, 17]. However, the ankle systolic blood pressure will be elevated in a noncompressible artery, and a false negative PAD diagnosis may occur when ABI alone is used for screening [22, 23]. Zahner et al. [24] reported that measuring the augmentation index of pulse waves in the radial artery helps improve PAD diagnosis. DM is closely associated with arterial stiffness [25, 26], and reportedly increases the risk of arterial stiffness by 1.8 times in a Chinese population [27]. In a study by Wukich et al., 42.7% of patients with DM and confirmed PAD had normal ABI values [28]. In contrast, a flattened pulse volume waveform in patients with DM was more closely associated with PAD than a low ABI value was, regardless of ankle arterial stiffness [29, 30]. The  %MAP represents the percentage difference between the mean and maximum amplitude of the ankle pulse volume waveform [12]. A flattened waveform resulting from an occluded artery will increase the  %MAP value, which is not strongly affected by noncompressible arteries [11, 30]. Therefore, the ankle  %MAP might be a sensitive indicator of an occlusive artery with a noncompressible pattern, which is frequently observed in patients with DM [31]. The advantage of  %MAP measurement in detecting arterial occlusion may explain why CVD-related mortality is markedly increased in patients with low ABI and high  %MAP in the present study. In line with our study,  %MAP was associated with CVD-related mortality in hemodialysis patients [32].

Arterial stiffness may involve extracellular destruction and cellular dysfunction of the vessel wall [33]. There are several potential mechanisms involved in the association between DM and arterial stiffness. First, glycation reactions during hyperglycemia can stiffen the arterial wall by inducing irreversible collagen cross-links [34]. Second, an overabundance of advanced glycation end products binding to their receptors on the vessel inner wall can activate a series of responses involving oxidation and inflammation [35]. An inflammatory process including the accumulation of macrophage and the activation of cell adhesion molecules can induce matrix metalloproteases to degrade the extracellular matrix and increase smooth muscle migration and proliferation [35, 36]. Oxidative stress can increase the vessel tone by impairing endothelial nitric oxide production and proliferating vascular smooth muscle cells [37–39]. Third, insulin resistance can increase the vessel tone by activating endothelin-1 and angiotensin II type 1 receptors [40, 41], and the Atherosclerosis Risk in Communities Study reported that insulin resistance was associated with arterial stiffness in patients with type 2 DM [42]. Arterial stiffness with high baPWV was also reported to be associated with new-onset DM [43]. Furthermore, a high prevalence of medial arterial calcification was reported in patients with DM [44, 45]. Noncompressible arteries are reportedly associated with mortality even in patients with high ABI [45, 46]. Therefore, the influence of noncompressible arteries on ABI must be considered in patients with type 2 DM.

In a previous study that enrolled 3004 Japanese participants, including 2598 (86%) with diabetes, the criterion of ABI < 0.9 predicted 20.4% of deaths within a mean follow-up duration of 4.4 years [47]. Notably, in the present study, the prediction of all-cause mortality was 21.1% with ABI alone and 38.0% with the combination of ABI and  %MAP criteria. Although the C index was only 0.62 for predicting all-cause mortality in the combined ABI and  %MAP model, it was still significantly higher than in the ABI alone model. Besides, the C index can reach to 0.72 by adding the common variables including age and sex in the model containing ABI and  %MAP. Furthermore, Cox regression analysis confirmed that ABI accompanied by  %MAP was superior to ABI alone in predicting the mortality of patients with type 2 DM.

The prevalence of PAD is increasing worldwide [48, 49]. Most patients with PAD are asymptomatic, but they have an elevated risk of mortality [48–50]. In Taiwan, annual screening for foot complications is recommended in the clinical guidelines and in the P4P program for patients with DM [14, 51]. In previous studies that have used the cutoff value of ABI ≤ 0.90, the prevalence of PAD in type 2 DM was approximately 10.0% in patients with a mean age of 63 years in Taiwan, 10.4% in Malay patients (mean age, 63 years) who lived in Singapore, and 9.5% in patients aged > 40 years in the US [52–54]. According to database of real-world clinical diagnosis, PAD was reported in 18.7% of patients with type 2 DM (mean age, 65 years) in the UK and in 13.6% of patients with type 2 DM (mean age, 66 years) in the US [55, 56]. In the present cohort, the prevalence of PAD was 8.4% when ABI ≤ 0.90 was the only criterion used, but the rate increased to 17.4% when the combination of ABI ≤ 0.90 and  %MAP > 45% was used. In the Taiwan National Health Insurance database, under 2.2% of patients with DM and age ≥ 65 years have a diagnosis of PAD, indicating that the condition is greatly underdiagnosed in clinical practice [57]. Thus, the use of ABI along with the automatically reported ankle  %MAP is an effective and convenient method for PAD screening and for prediction of mortality [10, 13].

The risk factors for abnormal ABI have been extensively investigated, but the specific risk factors for high  %MAP are still not clear [58, 59]. In the present study, the risk factors significantly associated with  %MAP at both ABI levels included age, CVD history, BMI, HbA1c, eGFR, UACR, baPWV, use of antiplatelet agents, type of oral antihyperglycemic drug taken, and type of hypertensive drug taken (Table 1). However, we did not include all cardiovascular risk factors in the present study; for example, it has previously been reported that higher HbA1c variability is associated with a higher  %MAP [19]. Furthermore, this study has several limitations. First, all participants were from a single teaching hospital, and the results may not be generalizable to all populations with type 2 DM. Second, this was a retrospective study, and so we could not control the risk factors and treatments received during the follow-up period. Third, we did not apply anatomical assessments to confirm the lesions of PAD in the present study with a primary endpoint of all-cause mortality. Finally, the cutoff value of 45% for  %MAP is based on the findings of previous studies [13]; we did not assess the normal range of  %MAP in the present study.

In conclusion, the use of  %MAP along with ABI appears to improve the prediction of all-cause mortality in patients with type 2 DM. The  %MAP is automatically reported during ABI measurement and therefore can be conveniently used to improved prognostic prediction in clinical practice.

## Data Availability

The datasets used and/or analyzed during the current study are available from the corresponding author on reasonable request.

## Abbreviations

%MAP: Percentage of the mean arterial pressure
ABI: Ankle-brachial index
AUC: Area under the receiver operating characteristic curve
baPWV: Brachial-ankle pulse wave velocity
CI: Confidence interval
CVD: Cardiovascular disease
DM: Diabetes mellitus
eGFR: Estimated glomerular filtration rate
HbA1c: Hemoglobin A1c
HDL: High-density lipoprotein
HR: Hazard ratio
IDI: Integrated discrimination improvement
NRI: Net reclassification improvement
P4P: Pay-for-performance
PAD: Peripheral artery disease
SD: Standard deviation
UACR: Urinary albumin-to-creatinine ratio
